# Supplementary material for: Wavelength Assignment in Hybrid Quantum-Classical Networks
Source: Sci Rep. 2018 Feb 22;8:3456. doi: 10.1038/s41598-018-21418-6 (PMC5823948; doi:10.1038/s41598-018-21418-6)
Supplement: Supplementary file 1 — Supplementary Information [file 41598_2018_21418_MOESM1_ESM.pdf]

Wavelength Assignment in Hybrid Quantum-Classical Networks

Sima Bahrani[1,2,*], Mohsen Razavi[1], and Jawad A. Salehi[2]

[1]School of Electronic and Electrical Engineering, University of Leeds, Leeds, LS2 9JT, UK
[2]Electrical Engineering Department, Sharif University of Technology, Tehran, Iran
[*]si.bahrani@gmail.com

Appendix A
Linear Approximation of Secret Key Rate

In this appendix, we analyze the secret key rate of a quantum channel and derive a linear approximation to it in certain regions of interest. From equation (11) in the main text, we can write

$$P(Y_0) = Q_1 x_1 - f Q_\mu x_2, \tag{1}$$

where

$$\begin{aligned} x_1(e_1) &= 1 - h(e_1), \\ x_2(E_\mu) &= h(E_\mu). \end{aligned} \tag{2}$$

To the first-order approximation, and for sufficiently small values of p, the entropy function $h(p)$ can be approximated as a linear function of p. In our case, the relevant values for e_1 and E_μ in (2) are expected to be small if we want to have positive key rates. For instance, if we assume that $\eta_d = 1$, $L = 0$, $e_d = 0$, and $f = 1$, and just increase Y_0 until $P(Y_0)$ becomes zero, we find that $e_1 < E_\mu < 0.0953$. That is, if we restrict ourselves to the regime in which key rates are positive, we can approximate x_1 by $ae_1 + b$ and x_2 by $kE_\mu + j$, for some constant parameters a, b, k, and j. We have verified that under the assumption of $e_1 < E_\mu < 0.0953$, the mean square error for these approximations is less than $1.89E - 4$. Now, if we substitute these linear approximations into (1) and use equations (12)-(15) in the main text, we obtain

$$P(Y_0) = UY_0 + V, \tag{3}$$

where

$$U = \frac{a}{2}\mu e^{-\mu} + b(1-\eta)\mu e^{-\mu} - \frac{k}{2} f - fje^{-\eta\mu}, \tag{4}$$

and

$$V = a\eta e_d \mu e^{-\mu} + b\eta\mu e^{-\mu} - kfe_d(1 - e^{-\eta\mu}) - fj(1 - e^{-\eta\mu}). \tag{5}$$

Finally, noting that, for $(p_{\rm dc} + p_m) \ll 1$, equation (18) in the main text can be approximated by $Y_0 \simeq 2p_{\rm dc} + 2p_m$, we obtain

$$P(Y_0) \approx 2Up_m + 2Up_{\rm dc} + V, \tag{6}$$

which means that the key rate can be written in a linear form versus p_m. Obviously, the above approximation holds when p_m is small enough that we still get positive key rates. Once this condition breaks down, as we saw in Fig. 7 in the main text, the optimal solution that maximizes the total key rate can differ from the one that minimizes the background noise.
